# Supplementary material for: The Use of Data from the Parkinson’s KinetiGraph to Identify Potential Candidates for Device Assisted Therapies
Source: Sensors (Basel). 2019 May 15;19(10):2241. doi: 10.3390/s19102241 (PMC6568025; doi:10.3390/s19102241)
Supplement: Supplementary file 1 [file sensors-19-02241-s001.pdf]

Supplementary Materials:

Table S1. The clinical and PKG characteristics of PwP in the Construction and Test Sets.

	Construction Set N = 112			Test Set N = 60		
	**CP**	**CN**	**Δ**	**CP**	**CN**	**Δ**
Age	63 (55–70)	72 (67–77)	9	64.7 (59.3–71)	67 (62–73)	-2.7
UPDRS I	6 (3–11)	7 (5.5–12.5)	-1	6 (3-10.5)	8.5 (3.7-16.7)	-1.5
UPDRS II	13 (10–20)	6 (3–11)	7	12 (10–18)	7.5 (5.2–13.7)	4.5
UPDRS III	25 (17–38)	27.5 (20–34.7)	-2.5	31 (20–34)	20 (17–35)	11
UPDRS IV	7 (4.5–9)	1 (0–4)	6	6 (2.5-–8.5)	1 (0–4)	5
UPDRS Total	52 (40–77)	44 (32.2–57.5)	8	53.5 (43.5–64)	38.5 (31–50.7)	5
Median BKS	20.7 (16–25.3)	23.7 (21.5–26.8)	-3	21.4 (17.2–25.4)	24.7 (23–28)	-3.3
PTB	36.9 (17.8–50.8)	42.8 (33.3–61.6)	-5.9	36.8 (18.2–56.4)	51.3 (44–72.2)	-14.5
Median DK	4.8 (2.3–14.9)	2.3 (1–4)	2.5	6.2 (2.6–12.8)	1 (0.7–2.8)	5.2
PTD	24.7 (10.2–48.5)	8.3 (3.7–15)	16.4	23.3 (10–46.4)	4.8 (1.7–11)	18.5
DBSS	99.5 (84.8–100)	7.9 (1.4–43)	91.6	95.4 (73.6–99.9)	18.7 (0.4–51.1)	76.7
Doses/day	5.5 (5–7)	4 (3–5)	1.5	5 (4–6)	4 (3–4)	1
PTT	0.8 (0.3–2.1)	0.8 (0.4-2)	0	1.1 (0.3–2.6)	1 (0.4–1.9)	0.1
PTI	4.4 (2–6.6)	4.3 (2.2–8.6)	0.1	3.2 (0.9–7.3)	5.8 (2.9–9.9)	-2.6
